# Integrating Age-Friendly Practices in Aging Service and Behavioral Health Systems: A Systems Change Initiative

**DOI:** 10.1093/geroni/igaf122.651

**Published:** 2025-12-31

**Authors:** Allison Gibson, Marla Berg-Weger, Julie Blanco, Britney Parson, Dana Silverblatt, Max Zubatsky, Dimitra Tziarli

**Affiliations:** Saint Louis University, Saint Louis University, Missouri, United States; Saint Louis University - - St Louis, MO, St. Louis, Missouri, United States; Illume - Behavioral Health Center of Excellence, St. Louis, Missouri, United States; Behavioral Health Network of Greater St. Louis, St. Louis, Missouri, United States; Behavioral Health Network of Greater St. Louis, St. Louis, Missouri, United States; Saint Lewis University, Saint Louis, Missouri, United States; Saint Louis University, St. Louis, Missouri, United States

## Abstract

The integration of aging services and behavioral health remains a significant challenge due to long-standing silos between the two systems. To address this, our team collaborated with 12 community-based aging-focused and behavioral health organizations in St. Louis, bringing together opportunities for greater collaboration. Through a series of meetings, these organizations shared their existing practices and services, allowing us to identify concrete actions to enhance integration. Each organization sets its own goals and timeline. Many organizations set goals to train staff and volunteers, adopt new assessment tools, and adjust referral processes to ensure more comprehensive care. As part of this effort, we developed an interview guide to assess the impact of these changes and better understand the barriers and facilitators to systems change. Our interview guide captures systems of change of these organizations: key insights into how organizations are implementing new strategies, what challenges they are facing, and what support they may need to sustain these efforts. Open-ended questions encouraged reflection on the effectiveness of training programs, changes in referral workflows, and overall improvements in service coordination. These agency interviews broaden the implications of these efforts, including the potential for scalability and sustainability beyond the initial implementation. In this presentation, we will discuss the best practices of system change, inform policy discussions of comprehensive care, and ensure that aging and behavioral health services are better integrated to serve the needs of the community.

